# Neuroprotective Effect of Bergamot Juice in 6-OHDA-Induced SH-SY5Y Cell Death, an In Vitro Model of Parkinson’s Disease

**DOI:** 10.3390/pharmaceutics12040326

**Published:** 2020-04-05

**Authors:** Nadia Ferlazzo, Santa Cirmi, Alessandro Maugeri, Caterina Russo, Giovanni Enrico Lombardo, Sebastiano Gangemi, Gioacchino Calapai, Vincenzo Mollace, Michele Navarra

**Affiliations:** 1 Department of Chemical, Biological, Pharmaceutical and Environmental Sciences, University of Messina, 98100 Messina, Italy; nferlazzo@unime.it (N.F.); scirmi@unime.it (S.C.); amaugeri@unime.it (A.M.); cate.russo.22@gmail.com (C.R.); gelombardo@unime.it (G.E.L.); 2 Department of Health Sciences, University “Magna Græcia” of Catanzaro, 88100 Catanzaro, Italy; mollace@unicz.it; 3 Fondazione “Prof. Antonio Imbesi”, 98100 Messina, Italy; 4 Department of Clinical and Experimental Medicine, University of Messina, 98100 Messina, Italy; gangemis@unime.it; 5 Department of Biomedical and Dental Sciences and Morphofunctional Imaging, University of Messina, 98100 Messina, Italy; gcalapai@unime.it

**Keywords:** *Citrus bergamia*, Parkinson’s disease, SH-SY5Y cells, natural products, 6-OHDA, bergamot

## Abstract

Much evidence suggests that both oxidative stress and apoptosis play a key role in the pathogenesis of Parkinson’s disease (PD). The present study aims to evaluate the protective effect of bergamot juice (BJ) against 6-hydroxydopamine (6-OHDA)- or H_2_O_2_-induced cell death. Treatment of differentiated SH-SY5Y human neuroblastoma cells with 6-OHDA or H_2_O_2_ resulted in cell death that was significantly reduced by the pre-treatment with BJ. The protective effects of BJ seem to correlate with the reduction of intracellular reactive oxygen species and nitric oxide generation caused by 6-OHDA or H_2_O_2_. BJ also attenuated mitochondrial dysfunction, caspase-3 activation, imbalance of pro- and anti-apoptotic proteins, MAPKs activation and reduced NF-ĸB nuclear translocation evoked by neurotoxic agents. Additionally, BJ exhibited excellent antioxidant capability in *cell-free* assays. Collectively, our results suggest that BJ exerts neuroprotective effect through the interplay with specific cell targets and its antioxidant activity, making it worthy of consideration for the management of neurodegenerative diseases.

## 1. Introduction

Parkinson’s disease (PD) is a progressive disorder of nervous system, affecting two to three percent of the population over 65 years old. Thus, PD represents the second-most common neurodegenerative illness with more than six million cases worldwide. It is defined by an incremental impairment of dopaminergic neurons localized in the substantia nigra, which is implied in motor control and reward. Although the true etiology of PD is yet under debate, several molecular events have been identified so far as important intermediaries of neuronal cell death, including oxidative/nitrosative stress, activation of neuroinflammatory processes, mitochondrial dysfunction and apoptotic cascade [1,2]. At present, no drug has been proved to exert clinically validated neuroprotective effect, showing their efficacy just on symptoms [3], so that development of more effective pharmacological strategies is highly desirable. Comprehensive research on novel neuroprotective drugs has proven that anti-inflammatory and antioxidant molecules from dietary sources may prevent and/or counteract neurodegenerative diseases, such as PD [4,5]. In this regard, nutraceuticals and food supplements have been shown to provide neuroprotection in several experimental models, and their use, as alternative to synthetic drugs or in combination with these, is justified by their capability of abolishing or, at least, mitigating the unwanted side effects of established therapies [5,6,7].

*Citrus bergamia* Risso et Poiteau (bergamot) is a small tree of the Rutaceae family, almost solely cultivated for its fruit, from which is extracted an essential oil widely used in the fragrance industry. However, it has been recently proposed that the bergamot essential oil (BEO) can exert benefits on health [8,9] thanks to its anti-infective [10], anti-cancer [11] and neuroprotective [12] effects. Instead, bergamot juice (BJ) was considered a byproduct until last decade, when its pharmacological activities have been described [13,14,15]. Many of those are due to its flavonoids, the most common polyphenolic compounds of human diet. Given the promising biologic activities of flavonoids [16,17,18,19], there is a great interest in their potential neuroprotective effect [20]. On this issue, several studies have suggested that *Citrus* flavonoids can prevent neurodegeneration, as well as other age-related conditions, and promote brain functions [7,21]. Evidence that the most representative flavonoids of *Citrus* fruit, including hesperidin, hesperetin and naringenin, can cross the brain-blood barrier [22], reinforces this assumption.

The 6-hydroxydopamine (6-OHDA) is a neurotoxin largely employed to reproduce experimental models of PD [23]. Its auto-oxidative metabolites cause cytotoxicity in various cell lines, including neuroblastoma cells [23], by generating hydrogen peroxide (H_2_O_2_), superoxide anion and hydroxyl radicals that, together with other reactive oxygen species (ROS), determine loss of mitochondrial membrane permeability, thus leading to the generation of oxidative stress. The mitochondrial impairment provokes release of cytochrome c and other pro-apoptotic proteins that activate downstream effectors such as caspase-3 that causes neuronal cell death.

On this basis, we investigated whether BJ protects differentiated SH-SY5Y cells from 6-OHDA- or H_2_O_2_-induced neurotoxicity, exploring its mechanism of action. This could give indications on the neuroprotective potential of BJ.

## 2. Materials and Methods

### 2.1. Drug

*Citrus bergamia* fruits were harvested in Bovalino (Reggio Calabria, Italy). Afterwards, they were hand-squeezed, and aliquots of juice were kept at −20 °C. For the experiments on cell cultures, the pH of BJ was arranged to 7.4, filtered and diluted in culture media to reach required concentrations prior to use. The chemical characterization of flavonoids present in BJ has been reported previously [24,25,26]. However, before starting this study, a qualitative and quantitative HPLC analysis was performed, confirming that the flavonoids composition corresponds to those already published [24,26]. Naringin, hesperetin, neohesperidin and neoeriocitrin are the most abundant flavonoids in the BJ tested in these studies.

### 2.2. Abiotic Assay

The antioxidant activity of BJ was assessed through the stable 2,2-diphenylpicrylhydrazyl (DPPH) radical assay, the reducing power determination and the oxygen radical absorbance capacity (ORAC) assay. Total phenolic content of BJ was measured through the Folin–Ciocalteu assay. All *cell-free* tests were performed following the procedure employed by Ferlazzo et al. [27].

### 2.3. Cell Culture

Experiments were carried out using the SH-SY5Y human neuroblastoma cell line (originally from ATCC, Rockville, MD, USA). Cells were differentiated in MEM/Ham’s F12 medium supplemented with 10-µM retinoic acid (RA; Sigma–Aldrich, Milan, Italy) for 5 days as reported by Condello et al. [28]. All reagents were from Gibco (Life Technologies, Monza, Italy).

### 2.4. Cytotoxicity Assay

Cell viability was assessed by the 1-(4,5-dimethylthiazol-2-yl)-3,5-diphenylformazan (MTT) test as reported [29]. The cells were plated into 96-well plates (5 × 10^4^ cells/well) and 24 h later were treated with BJ 0.5% or 1% for 1 h. Then, 6-OHDA (50 or 100 µM; Sigma–Aldrich) or H_2_O_2_ (50–150 µM; Sigma-Aldrich) were added for additional 24 h. The absorbance was recorded at 570 nm (reference at 690 nm) by a microplate spectrophotometer.

Cell death was assessed by the trypan blue (0.4% *w*/*v*; TB) exclusion test. Cells were seeded onto 6-well plates at a density of 10 × 10^3^ cells/well for 24 h, and then treated with BJ 0.5% or 1% for 1 h prior to be incubated with either 6-OHDA (50 or 100 µM) or H_2_O_2_ (50 or 100 µM) for further 24 h. Then, cells were trypsinized, centrifuged, re-suspended in a known volume of PBS and stained with the trypan blue dye, before proceeding with cell count [30,31].

### 2.5. Detection of Apoptosis by Annexin-V FITC Staining

AnnexinV/PI staining was employed to discriminate cells among living, apoptotic and necrotic. Cells were seeded in 6-well plates at a density of 5 × 10^5^ cells/well and left for 24 h. Afterward, the cultures were pre-treated for 1 h with BJ (0.5% and 1%), and then exposed to 50-µM 6-OHDA. After 24 h of incubation, the cells were processed as reported [19]. Finally, specimens were run on NovoCyte Flow Cytometer Systems (ACEA Biosciences Inc., San Diego, CA, USA).

### 2.6. Spectrofluorimetric Determination of ROS and Δψm

As oxidative stress biomarkers, ROS and mitochondrial membrane potential (Δψm) were measured by fluorometric methods. In both assays, the cells were seeded onto 96-well plates (5 × 10^4^ cells/well), and the following day, they were pre-treated with BJ (0.5% and 1%) for 1 h and then exposed to 6-OHDA (50 µM) or H_2_O_2_ (100 µM) for additional 6 h.

ROS were quantified using 2′,7′-dichlorodihydrofluorescein diacetate (DCFH-DA 25 µM; Sigma–Aldrich) as reported [12]. The fluorescence was recorded by a microplate reader (POLARstar Omega, BMG Labtech, Ortenberg, Germany) at 485 nm excitation and 535 nm emission.

Changes in Δψm were estimated by measuring Rhodamine 123 incorporation (R123; Sigma-Aldrich), as reported by Condello et al. [28]. The intensity of fluorescence was recorded at 488 nm excitation and 525 nm emission.

### 2.7. Caspase-3 Enzymatic Activity

Caspase enzyme activity was measured using a commercial kit (AbCam, Cambridge, UK). SH-SY5Y cells were differentiated in 100 mm petri dishes (1.5 × 10^6^ cells), and then treated with 0.5% or 1% BJ for 1 h, prior to be incubated with 50-µM 6-OHDA for 6 h. Then, according to the manufacturer’s instructions, cells were lysed, centrifuged and on the supernatant was carried out the analysis. The absorbance was measured at a wavelength of 405 nm by a microplate spectrophotometer.

### 2.8. Western Blot

SH-SY5Y cells (1.5 × 10^6^ cells) were differentiated, pre-treated with 0.5% or 1% BJ for 1 h and then incubated with 50-µM 6-OHDA for additional 6 or 24 h. Total cellular lysates were prepared, quantified and electrophoresed (30 µg/lane) as reported by Celano et al. [32]. The membranes were blocked with 5% nonfat milk and then incubated overnight at 4 °C with the following antibodies: rabbit anti-phospho p44/42 MAPK (Thr 202/Try 204) and anti-p44/42 MAPK (Cell Signaling Technology, Beverly, MA, USA); rabbit anti-phospho p38 and rabbit anti-p38 (AbCam); rabbit anti-iNOS and nNOS (Becton Dickinson, Franklin Lakes, NJ, USA); mouse anti-p53 (AbCam); mouse anti-Bax and anti-Bcl-2 (Thermo Fisher Scientific, Waltham, MA, USA); rabbit anti-β-actin (Cell Signaling Technology). Secondary goat HRP-conjugated anti-mouse or anti-rabbit IgG antibody (AbCam) were incubated at room temperature for 2 h. A representative immunoblot image of three independent experiments for each target is shown. Autoradiographic bands were quantified by ImageJ software and normalized for β-actin levels.

### 2.9. Evaluation of NF-ĸB Activation

The presence of NF-ĸB DNA binding activity was assessed by a gel electromobility shift assay (EMSA) kit (Panomics, Inc., Fremont, CA, USA) [25,33]. To this aim SH-SY5Y were seeded in 100 mm Petri dishes (1.5 × 10^6^ cells) and differentiated as above described. The cells were treated with BJ 0.5% or 1% for 24 h, and then incubated with 50-µM 6-OHDA for 6 h. Afterwards, the nuclear proteins were extracted from cold PBS washed cells using a commercial kit for nuclear extraction (Panomics), observing manufacturer’s guidelines. First, protein amounts were determined employing a protein assay commercial kit (Bio-Rad Laboratory, Hercules, CA, USA). Then, DNA-protein binding reaction was carried out in supplied binding buffer containing 1 µL of probe (NF-κB 5′ AGTTGAGGGGACTTTCCCAGGC 3′), 4 µg of nuclear extracts and 1 µL poly(dI-DC) at 15 °C for 30 min. After resolving complexes on a 6% non-denaturing acrylamide gel, these were transferred onto positively charged nylon membranes, and cross-linked at 120 mJ/cm^2^ for 1 min using a UV-light cross-link instrument (UV Stratalinker 1800, Stratagene, San Diego, CA, USA). DNA-protein binding was detected using streptavidin-labeled horseradish peroxidase (HRP) conjugate with a chemiluminescent detection system.

### 2.10. Determination of NO Accumulation in SH-SY5Yculture Supernatant

Nitric oxide (NO) production was assayed by a colorimetric kit (Sigma–Aldrich). In a 6-well plate (5 × 10^5^ cells/well) the differentiated cells were pre-incubated with BJ for 1 h, and then treated with 50-µM 6-OHDA for 24 h. The supernatants were collected and processed following manufacturer’s guidelines. Absorbance was spectrophotometrically quantified at a wavelength of 540 nm by a microplate spectrophotometer.

### 2.11. Statistical Analyses

One-way analysis of variance (ANOVA) was employed to analyze data. Multiple comparisons of the means of the groups were performed by the Tukey–Kramer test (GrafPAD Software for Science, version 7.0, Graphpad Software, Inc., San Diego, CA, USA).

## 3. Results

### 3.1. Antioxidant Activity of BJ in Abiotic Models

**Figure 1:**
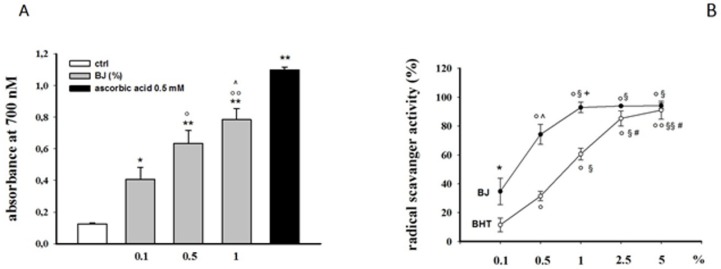
Antioxidant property of bergamot juice (BJ). Evaluation of reducing power (**A**) and DPPH scavenging activity (**B**) of BJ. The assays were carried out in triplicate and repeated three times. The results are expressed as mean value ± S.E.M. Different solutions of ascorbic acid (0.5 mM; **A**) or dibutylhydroxytoluene (BHT) (0.1–5% *w*/*v*; **B**) were used as reference. * *P* < 0.05 and ** *P* < 0.01 vs. ctrl, ° *P* < 0.05 and °° *P* < 0.01 vs. BJ 0.1%, ^ *P* < 0.05 vs. BJ 0.5% (**A**); ° *P* < 0.01 vs. BJ 0.5% and BHT 0.5%, °° *P* < 0.001 vs. BHT 0.5%, ^§^ *P* < 0.01 vs. BJ 0.5% and BHT 0.5%, ^§§^ *P* < 0.001 vs. BHT 0.5%, ^#^ *P* < 0.001 vs. BHT 1%, * *P* < 0.01 vs. BHT 0.1%, ^ *P* < 0.01 vs. BHT 0.5%, ^+^ *P* < 0.01 vs. BHT 1% (**B**).

Both the antioxidant and the radical scavenging properties of BJ have been demonstrated using numerous *cell-free* tests. The total phenolic amount, evaluated by Folin–Ciocalteu method, was 2.23 ± 0.04 mg GAE/mL of BJ, that, along with the results of the Reducing Power test (Figure 1A), suggested the antioxidant capability of BJ in abiotic models. Both Folin–Ciocalteu and Reducing Power assays are electron/transfer-based tests, performed in high and low pH values, respectively. In abiotic, low pH may hinder the activity of antioxidants with a phenolic moiety, due to protonation, while high pH could cause the opposite, due to dissociation [15]. Consequently, it is reasonable to assess the antioxidant capability of polyphenolic compounds by a series of abiotic tests. BJ also demonstrated a noticeable capability to dampen free radicals in the DPPH test (BJ and dibutylhydroxytoluene (BHT) IC_50_ was 0.4% ± 0.012 and 0.8% ± 0.03, respectively; Figure 1B) and a high ORAC value (4300 ± 425 µmol/TE/L juice) strengthens the results mentioned above.

### 3.2. BJ Prevents 6-OHDA- or H_2_O_2_-Induced SH-SY5Y Cell Death

**Figure 2:**
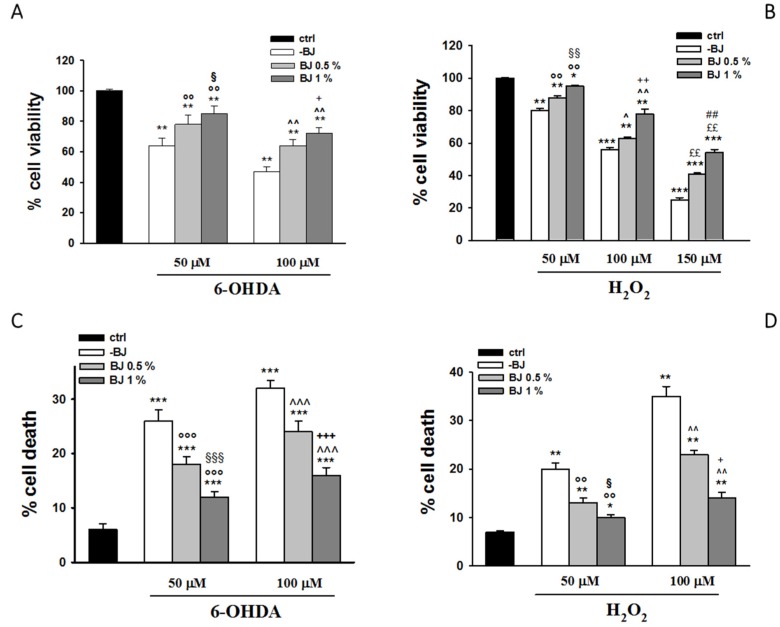
Effect of BJ on cytotoxicity induced by stressors. Cell viability was detected with the 1-(4,5-Dimethylthiazol-2-yl)-3,5-diphenylformazan (MTT) assay (**A**,**B**). The change of viability was determined as percentage of viable cells in treated cultures compared to those in untreated ones. Cell death was evaluated by the trypan blue (TB) assay and expressed as percentage of non-viable (blue stained) vs. total cells counted (**C**,**D**). Results are showed as means ± S.E.M. from three independent experiments performed eight-fold (MTT) or in triplicate (TB). * *P* < 0.05, ** *P*< 0.01 and *** *P* < 0.001 vs. ctrl; °° *P* < 0.01 and °°° *P* < 0.001 vs. 6-hydroxydopamine (6-OHDA) or H_2_O_2_ 50 µM; ^§^ *P* < 0.05, ^§§^ *P* < 0.01 and ^§§§^ *P* < 0.001 vs. 6-OHDA 50 µM or H_2_O_2_ + BJ 0.5%; ^ *P* < 0.05, ^^ *P* < 0.01 and ^^^ *P* < 0.001 vs. 6-OHDA or H_2_O_2_ 100 µM; ^+^ *P* < 0.05, ^++^ *P* < 0.01 and ^+++^ *P* < 0.001 vs. 6-OHDA 100 µM or H_2_O_2_ + BJ 0.5%; ^££^ *P* < 0.01 vs. H_2_O_2_ 150 µM; ^##^ *P* < 0.01 vs. H_2_O_2_ 150 µM + BJ 0.5%.

With a view to assess the potential neuroprotective capability of BJ, the cells were pre-incubated with BJ (0.5% or 1%) for 1 h and next exposed to 6-OHDA (50 or 100 µM) for another 24 h, prior to evaluate the cell viability. As displayed in Figure 2A, when the cells were incubated with the lowest concentration of 6-OHDA, viable cells were 65% respect to control cells (*P* < 0.01). The pre-treatment with BJ 0.5% or 1% significantly restored cell viability up to 78% and 85%, respectively (*P* < 0.01 respect to 6-OHDA-injured cells). The exposure to 100-µM 6-OHDA reduced cell viability up to 50% (*P* < 0.01), whereas the pre-treatment with BJ was able to prevent cell death. Indeed, BJ 0.5% or 1% significantly counteracted the cell death evoked by the highest concentration of 6-OHDA up to a 64% and 72% of cell viability, respectively (*P* < 0.01 versus 6-OHDA-injured cells; Figure 2A).

In parallel experiments, the cells were exposed to 50, 100 and 150-µM H_2_O_2_ for 24 h, provoking a reduction of cell viability from 20% to 75% (*P* < 0.01 for 50-µM H_2_O_2_ and *P* < 0.001 for 100 and 150-µM H_2_O_2_ versus respective control cells, Figure 2B). The pre-treatment (1 h) with BJ 0.5% or 1% prevented cell death evoked by H_2_O_2_ (*P* < 0.05 and *P* < 0.001 versus H_2_O_2_-incubated cells, respectively).

Data of MTT assays are in line with those obtained by the TB tests (Figure 2C,D).

Since 6-OHDA at both 50 µM and 100 µM was able to significantly reduce cell viability, even though in a slightly different measure, further experiments were carried out using its lowest concentration, while H_2_O_2_ was used at 100-µM concentration.

### 3.3. BJ Reduces Intracellular ROS Accumulation and Restores Impaired Δψm Elicited by 6-OHDA or H_2_O_2_ Exposure

**Figure 3:**
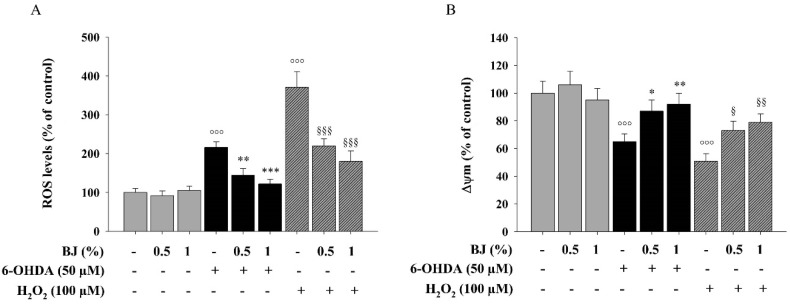
BJ attenuates the accumulation of reactive oxygen species (ROS) and the loss of Δψm induced by both 6-OHDA and H_2_O_2_. (**A**) ROS generation was measured by a fluorescent method, using DCFH-DA as probe. (**B**) Variations of Δψm were assessed using the cationic fluorochrome R123. Results are reported as percentage of the levels detected in untreated cells. Data are shown as the mean ± S.E.M. of three experiments. °°° *P* < 0.001 vs. control; *, ** and *** *P* < 0.05, *P* < 0.01 and *P* < 0.001 vs. 6-OHDA-treated cells, respectively; ^§^, ^§§^ and ^§§§^ *P* < 0.05, *P* < 0.01 and *P* < 0.001 vs. H_2_O_2_-treated cells, respectively.

Since the rise of ROS is involved in many pathogenic processes, including PD, we investigated the intracellular ROS level employing the fluorescent probe DCFH-DA. Exposure of SH-SY5Y cells to 50-µM 6-OHDA or 100-µM H_2_O_2_ for 6 h induced a significant intracellular ROS accumulation by 2.2-fold and 3.7-fold, respectively, when compared to the controls (*P* < 0.001; Figure 3A). Pre-treatment with BJ 0.5% and 1% for 1 h significantly counteracted 6-OHDA-induced ROS generation of 34 (*P* < 0.01) and 44% (*P* < 0.001), respectively (Figure 3A). In the same manner, BJ 0.5% and 1%, reduced ROS levels in H_2_O_2_-exposed cells by 40% and 51%, respectively (*P* < 0.001; Figure 3A).

In addition, exposure of SH-SY5Y cells to 50-µM 6-OHDA or 100-µM H_2_O_2_ for 6 h significantly affected Δψm, that, in comparison to control cells, decreased by 35% and 50%, respectively (*P* < 0.001). The reduction of Δψm induced by 6-OHDA or H_2_O_2_ was prevented by both BJ 0.5% and 1% (*P* < 0.05 and *P* < 0.01, respectively; Figure 3B).

### 3.4. BJ Reduces the Apoptotic Cell Death Induced by 6-OHDA

**Figure 4:**
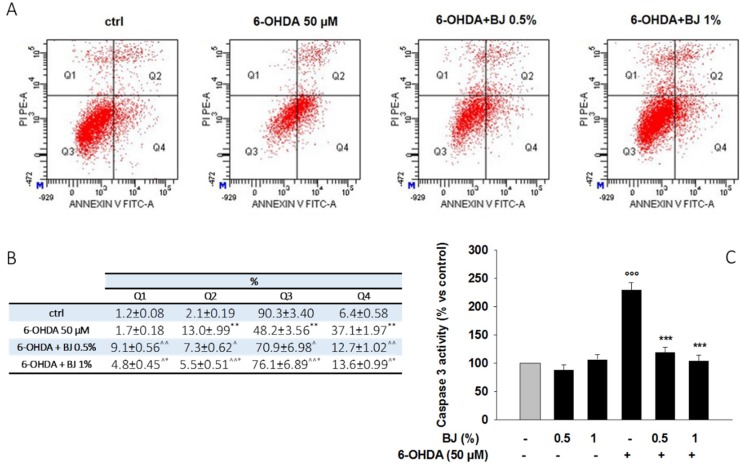
BJ attenuates 6-OHDA-induced apoptosis. (**A**) Evaluation of apoptosis was performed through the Annexin V/PI test. Representative Annexin V vs. PI dot plots are shown. Q1, necrotic cells; Q2, late apoptosis cells; Q3, viable cells; Q4, early apoptosis cells. (**B**) The table shows the percentage of cells in each quadrant, representing the mean ± S.E.M. of three different experiments. ** *P* < 0.01 vs. control; ^ *P* < 0.05 and ^^ *P* < 0.01 vs. 6-OHDA; ° *P* < 0.05 vs. 6-OHDA+BJ 0.5%. (**C**) Data of caspase-3 activity are presented as the mean of three experiments ± S.E.M. °°° *P* < 0.001 vs. control; *** *P* < 0.001 vs. 6-OHDA.

The cytoprotective effect of BJ was also assessed by cytofluorimetric analysis through the Annexin V fluorescein isothiocyanate (FITC)/propidium iodide (PI) assay. As shown in Figure 4A,B, the incubation of SH-SY5Y cells with 50-µM 6-OHDA for 24 h increased the percentage of cells in early (37.1%, AnnexinV+/PI-) and late (13%, AnnexinV+/PI+) apoptosis. The pre-treatment with BJ reduced the number of cells undergoing apoptosis, with 12.7% and 7.3% of early and late apoptosis when the cells where pre-incubated with BJ 0.5% for 1 h. Similarly, in presence of BJ 1%, the percentage of cells in both early and late apoptosis was of 13.6% and 5.5%, respectively (Figure 4A,B).

The occurrence of apoptosis in 6-OHDA-treated cells was confirmed by the results of the caspase-3 activity assay. The exposure to 50-µM 6-OHDA for 6 h increased caspase-3 activity of SH-SY5Y cells compared to the unexposed ones (*P* < 0.001; Figure 4C). Pre-treatment with BJ (0.5% or 1%; 1 h) inhibited the activity of caspase-3 brought by 6-OHDA (*P* < 0.001), while BJ alone had no effect on this enzymatic activity.

**Figure 5:**
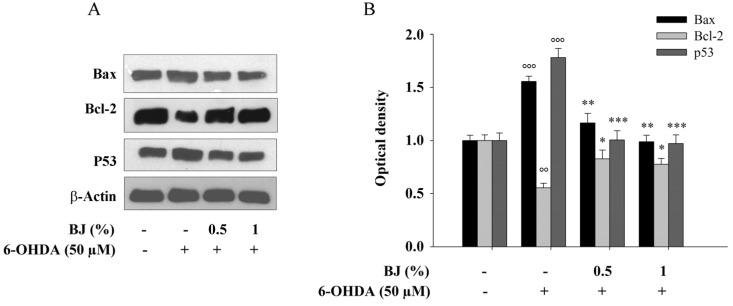
Protective effect of BJ on apoptotic-related proteins. SH-SY5Y cells were treated as reported above. Then, p53, Bax and Bcl-2 proteins levels were measured by western blotting. Representative immunoblots are displayed (**A**). Densitometric analysis of three independent blots (mean ± SEM) is depicted (**B**). Proteins amount were extrapolated as the values found in the untreated cells which were arbitrarily expressed as 1. °° *P* < 0.01 and °°° *P* < 0.001 vs. control cells; *, ** and *** *P* < 0.05, *P* < 0.01 and *P* < 0.001 vs. 6-OHDA-treated cells, respectively.

Furthermore, compared to control cells, the incubation of SH-SY5Y cells with 50-µM 6-OHDA for 24 h significantly enhanced the levels of the pro-apoptotic proteins Bax and p53 up to 1.5- and 1.7-fold (*P* < 0.001), respectively, as well as decreased those of the anti-apoptotic Bcl-2 up to 0.5-fold (*P* < 0.01). These outcomes were significantly counteracted by the pre-exposure to BJ at both 0.5% and 1% concentrations (*P* < 0.05 for Bcl-2, *P* < 0.01 for Bax and *P* < 0.001 for p53; Figure 5A,B).

### 3.5. Effect of BJ on 6-OHDA-Induced MAPKs Activation

**Figure 6:**
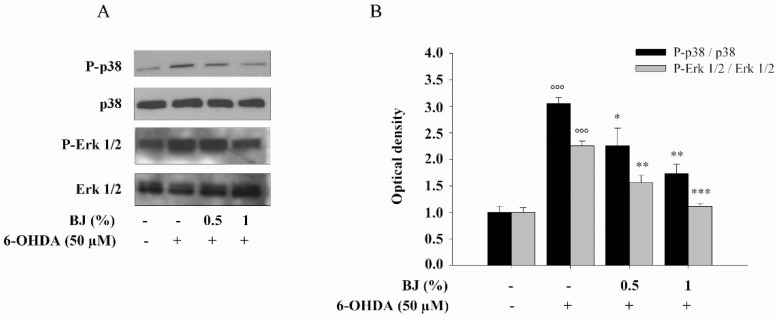
BJ reduces the phosphorylation of mitogen-activated protein kinases (MAPKs) induced by 6-OHDA. 6-OHDA induces the phosphorylation of Erk1/2 and p38 MAPKs in SH-SY5Y cells that was prevented by the pre-treatment with BJ. A representative immunoblot is depicted (**A**). The densitometric analysis of three separate blots (mean ± S.E.M.) is showed (**B**). Protein levels are extrapolated as values detected in control cells, which are arbitrarily assigned as 1. °°° *P* < 0.001 vs. control; *, ** and *** *P* < 0.05, *P* < 0.01 and *P* < 0.001 vs. 6-OHDA-treated cells.

We further examined the involvement of p38 and extracellular signal-regulated 1 and 2 (ERK1/2), two mitogen-activated protein kinases (MAPKs), which are related to oxidative stress-induced cell death and survival. Figure 6 shows that incubation of differentiated SH-SY5Y cells with 6-OHDA (50 µM) for 6 h enhanced the phosphorylation of both ERK1/2 and p38 (up to 2.2 and 3-fold versus control, respectively; *P* < 0.001). Treatment with neuroprotective concentration of BJ prevented both p38 (*P* < 0.05 and *P* < 0.01 for BJ 0.5% and 1%, respectively) and ERK1/2 (*P* < 0.01 and *P* < 0.001 for BJ 0.5% and 1%, respectively) phosphorylation induced by 6-OHDA (Figure 6A, B), without affecting total levels of the inactive forms.

### 3.6. BJ Reduces NF-κB Activation Elicited by 6-OHDA

**Figure 7:**
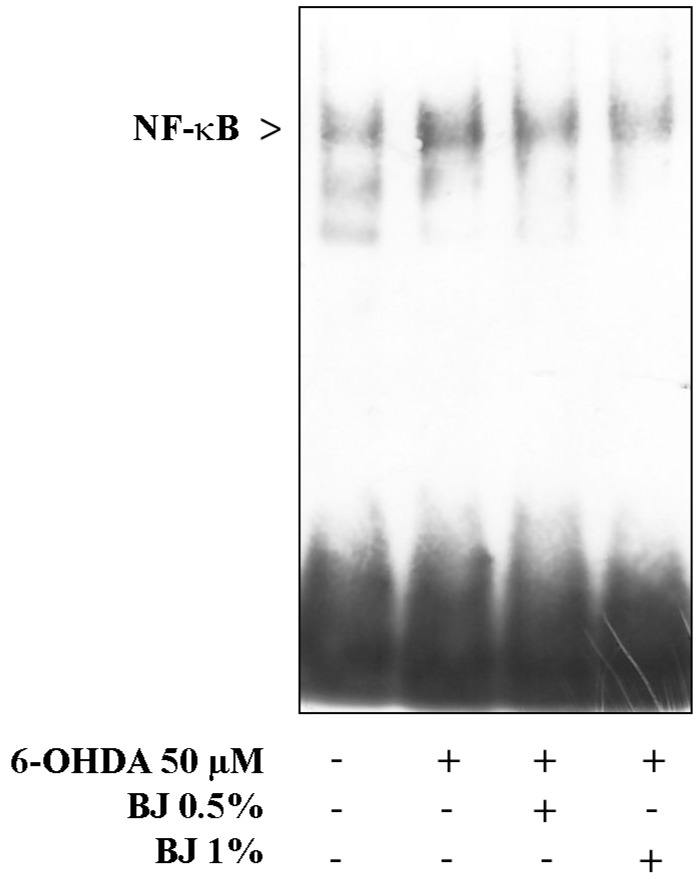
BJ reduces the activation of NF-ĸB induced by 6-OHDA. The gel electromobility shift assay (EMSA) analysis showing the nuclear translocation of NF-ĸB was performed after the exposure of differentiated cells to 6-OHDA subsequent to a pre-treatment with BJ. A representative image of two independent experiments is displayed.

In order to better clarify the mechanism of neuroprotection exerted by BJ, we investigated whether BJ prevents the activation of the nuclear transcription factor-κB (NF-κB) by EMSA analysis. Cell treatment with 50-µM 6-OHDA elicited NF-κB nuclear translocation within 6 h of exposure, while 1 h of pre-treatment with BJ prevented the activation of this nuclear factor. As shown in Figure 7, both BJ concentrations were able to reduce the nuclear translocation of NF-κB evoked by 6-OHDA.

### 3.7. BJ Reduces the Cellular Production of NO and the Levels of nNOS and iNOS

**Figure 8:**
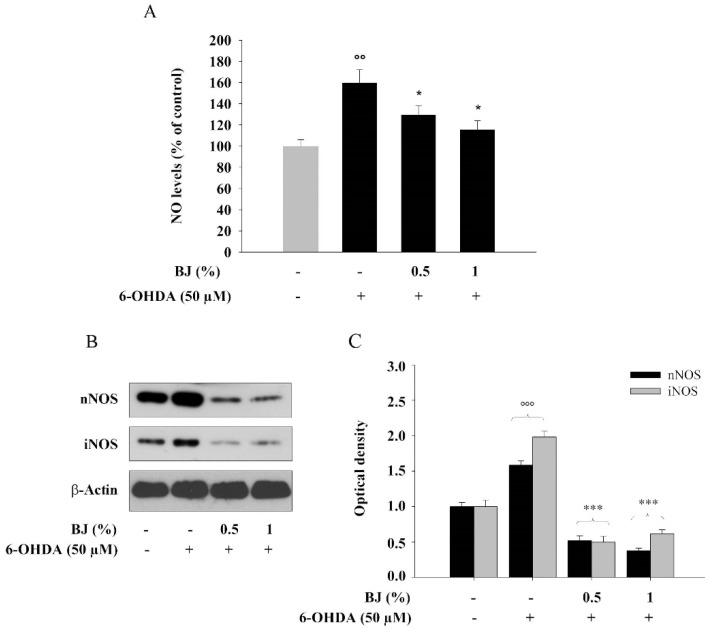
BJ prevents the 6-OHDA-induced NO release and reduces the expression of nNOS and iNOS. (**A**) Differences in NO production found in cells either or not pre-treated with BJ and then exposed to the 6-OHDA are reported as percentage of NO value detected in treated cells compared to those found in untreated ones. The levels of NO were measured by a colorimetric assay. Results are expressed as means ± S.E.M. from three independent experiments. (**B**) Levels of nNOS and iNOS proteins from cell cultures treated as described above, were determined by western blot analyses. Representative immunoblots are shown. (**C**) Densitometric analysis of blots from three different experiments (mean ± S.E.M.) is presented. °°° *P* < 0.001 and °° *P* < 0.01 vs. control; * and *** *P* < 0.05 and *P* < 0.001 vs. 6-OHDA treated cells, respectively.

Exposure of differentiated SH-SY5Y cells to 50-µM 6-OHDA for 24 h led to 50% increase of NO production (*P* < 0.01; Figure 8A), that was prevented by 1 h of pre-treatment with BJ (*P* < 0.05 compared to 6-OHDA-treated cells; Figure 8A), while BJ alone at both 0.5% and 1% concentrations had no effect on NO production (data not shown). Data of western blot analysis strengthen these findings, showing that BJ at both tested concentrations reduced significantly the rise of both nNOS and iNOS expression caused by 6-OHDA (*P* < 0.001; Figure 8B,C). These results indicate that the BJ-protective effect against 6-OHDA-induced SH-SY5Y cell death, at least in part, can occur preventing the rise of intracellular NO levels.

## 4. Discussion

This study, for the first time, shows the neuroprotective potentiality of BJ. We investigated the effect of this freshly squeezed *Citrus* juice against 6-OHDA-induced toxicity in differentiated human neuroblastoma SH-SY5Y cells, an experimental model extensively used to resemble PD in vitro [23].

Although neuropathological features of PD are well described, to date, the exact etiology of PD is not completely understood, whereas 6-OHDA may be considered one of the main responsible of its pathogenesis. Indeed, it has been detected in both brain and urine of PD patients [34] and it is capable to produce a toxicity similar to the neuropathological and biochemical characteristics of PD [23]. The 6-OHDA is a highly oxidizable dopamine analog that may be oxidized by molecular oxygen generating large amount of ROS, that in turn determine mitochondrial impairment, lipid peroxidation, DNA damage and cell death. Consistently, several studies suggest that increased ROS and mitochondrial impairment act in the pathogenesis of PD [18]. Therefore, novel pharmacological strategies based on antioxidant molecules are desirable, in order to reduce the progression of dopaminergic cell death observed in patients with PD and to obtain a better management of them.

Recently, we demonstrated that BJ, along with its flavonoid-rich extract (BJe), possesses antioxidant [27,35] and anti-inflammatory [33,36,37,38,39,40] activities. We further demonstrated the antimicrobial effect of BJe [41], as well as its anti-cancer properties, both in vitro [24,25,42] and in vivo [26,43]. On the other hand, the beneficial effects of *Citrus* fruits and their juice against degenerative conditions are well-known [10,13,37,44,45,46,47,48].

In this paper, we first documented the antioxidant activity of BJ in *cell-free* assays and, afterwards, we demonstrated its ability to reduce ROS levels produced by both 6-OHDA and H_2_O_2_. Since 6-OHDA-induced ROS trigger mitochondrial membrane damage, resulting in the collapse of Δψm, leading to apoptotic cell death [49], the blockage of ROS generation may represent an important intervention to protect neurons. The neuronal cell death can also be due to the overproduced H_2_O_2_, as occurring in pathological process of acute and chronic neuronal toxicity, including PD. The free radical-scavenging activity exerted by BJ can be responsible for the restoration of Δψm, that we observed in the SH-SY5Y cultures pre-treated with the juice and then exposed to the two agents causing oxidative stress. Breaking this vicious circle, BJ protects the SH-SY5Y cells from the oxidative cell death triggered by either 6-OHDA or H_2_O_2_ that occurs by both apoptosis and necrosis.

Apoptosis is finely controlled by several factors, among which tumor suppressing and inducing genes, which could promote cell survival or induce apoptosis. It is known that high levels of ROS cause apoptosis, triggered by mitochondrial alterations and release of pro-apoptotic factors [50]. In turn, ROS accumulation may be the result of mitochondrial dysfunction and can be involved in cell death [51]. Moreover, ROS mediate intracellular signaling cascades and the activation of some apoptotic factors, such as proteins of the Bcl-2 family [18]. In this study, we demonstrated that BJ inhibits the activation of caspase-3 induced by 6-OHDA, as well as reduces the levels of the pro-apoptotic protein Bax, along with increasing those of the anti-apoptotic Bcl-2. On these bases, we can assume that, at least in part, BJ prevents the apoptosis elicited by 6-OHDA through the protection of mitochondria.

The MAPKs pathways have a prominent role in regulating cellular processes such as proliferation, differentiation and adaptation, and are involved in the pathogenesis of PD [52]. Once activated, ERK, JNK and p38 phosphorylate several transcription factors and cytosolic proteins, with a subsequent augmentation of their transcriptional activities and activation of dependent genes [52]. Moreover, it has been reported that MAPKs are necessary for 6-OHDA-induced apoptosis [53]. In accordance with previous reports, we observed that treatment with 6-OHDA increased phosphorylation of ERK1/2 and p38 in SH-SY5Y cells, confirming a link between ROS generation by 6-OHDA and initiation of MAPKs signaling. The pre-treatment of neuroblastoma cells with BJ reduced the ERK1/2 and p38 phosphorylation, indicating that the neuroprotective effects of BJ against 6-OHDA toxicity is related to the modulation of MAPKs pathways.

One target of activated MAPKs is the nuclear transcription factor NF-ĸB which, from the inactive state in the cytosol, following phosphorylation of its inhibitory subunits, translocates into the nucleus, where acts as a transcriptional regulator of other proteins. It has been reported that the activation of NF-ĸB is involved in the pathogenesis of several neurodegenerative disorders and increased in dopaminergic neurons of PD patients [54]. Several in vitro studies reported that the activation of NF-ĸB plays a pivotal role in 6-OHDA induced cell death. Authors reported that some pharmacological agents possess neuroprotective effect due to their ability to block NF-κB activation, suggesting its pro-apoptotic role in PD [55,56,57], thus being a possible target to counteract neurodegenerative diseases. In this regard, we investigated the effects of BJ on NF-ĸB activation induced by 6-OHDA, and we found that BJ prevented the NF-ĸB activation by inhibiting its nuclear translocation. Once activated, NF-κB increases the expression of different genes implied in either cell death or survival, including p53 and c-Myc [58]. P53 is implied in apoptotic death of dopaminergic neurons, and its increased expression has also been reported in cellular model of PD [59]. In addition, p53 is a positive transcriptional activator for Bax and a negative one for Bcl-2 and Bcl-XL [60]. In our study, western blot analysis displayed that p53 increased significantly in cells treated with 6-OHDA and the levels were restored by the pre-treatment with BJ, suggesting that both NF-κB and p53 take part the mechanism through which BJ acts against 6-OHDA injury.

Nitric oxide (NO) is a signaling molecule in the biologic system which is thought to be also a neurotoxin. In this regard, its excessive production in brain causes both neuronal damage and death. It has been demonstrated that NO is implicated in the pathogenesis of neurodegenerative diseases, including PD [61]. There are three known isoforms of nitric oxide synthase (NOS) in the mammals: neuronal NOS (nNOS), inducible NOS (iNOS) and endothelial NOS (eNOS). Each isoform is recognized to be strictly related to the pathogenesis of PD. Here, we reported that the expression of nNOS and iNOS enhanced after the treatment of differentiated SH-SY5Y with 6-OHDA, as well as increased the production of NO. Of note, BJ decreased the release of NO and both nNOS and iNOS protein levels. Considering that iNOS is a downstream target of NF-κB, the changes in iNOS levels observed in this study could be derived from the reduced activity of NF-ĸB induced by BJ.

## 5. Conclusions

To summarize the results of our in vitro study, we showed that BJ markedly inhibited 6-OHDA-induced apoptosis in differentiated human neuroblastoma SH-SY5Y cells, through mitigating the ROS and NO generation, the mitochondrial dysfunctions and the imbalance of Bcl-2 family proteins, as well as reducing the activation of MAPKs and the nuclear translocation of NF-ĸB. To the best of our knowledge, this is the first report showing the neuroprotective effect of BJ, exerted by mechanisms involving the interplay with specific cell targets and its antioxidant activity, making it worthy of consideration in the field of neurodegenerative diseases.
